# Design of a novel cell-permeable chimeric peptide to promote wound healing

**DOI:** 10.1038/s41598-018-34684-1

**Published:** 2018-11-02

**Authors:** Mareike Horn, Ines Neundorf

**Affiliations:** Department of Chemistry, Biochemistry, University of Cologne, Zuelpicher Str. 47a, 50674 Cologne, Germany

## Abstract

Biological membranes are impermeable to almost all compounds having a molecular weight greater than 500 Da. Recently, cell penetrating peptides (CPPs) as delivery vehicles have attracted great interest in the medical sector for the development of novel therapeutic agents or cosmetic products. Herein, a wound healing promoting sequence, namely Tylotoin, was covalently coupled with a cell penetrating peptide to improve the delivery of Tylotoin across cellular membranes. Indeed, internalization studies indicated that the cellular uptake of these novel peptide conjugates into keratinocytes was significantly improved accompanied by good tolerability. In a scratch wound closure assay used to investigate the wound healing capability, the most promising novel peptide chimera (Tylotoin-sC18*) was found to promote the migration of keratinocytes indicating that the fusion to Tylotoin did not cause any loss in its activity. Even more, proliferative effects on keratinocytes were observed, an important step during the wound healing process. Still more encouraging is the capability of Tylotoin-sC18* to exhibit strong antimicrobial activities since the process of wound healing is often affected by bacterial infections. Owing to their multiple functions, the novel peptide chimera may have potential as future agents for the treatment of infected wounds.

## Introduction

Efficient wound treatment remains one of the most prevalent healthcare issue as thousands of patients suffer annually from different kinds of skin damage, burns or diseases^1–3^. Thus, the delivery of therapeutic active molecules through the skin is receiving considerable scientific attention during the last years. However, since the skin serves as a protective barrier against external chemical, mechanical, microbial and physical influences^4^, it is very challenging to overcome the cellular membrane impermeability. In this regard, the discovery of so-called cell penetrating peptides (CPPs) in the last decade has been described as promising approach for the delivery of large molecules across biological membranes^5^.

In general, CPPs are relatively short, usually less than 40 amino acids in length, and commonly feature a highly positive net charge. Likewise they are able to carry electrostatically or covalently bound biologically active cargoes across cellular membranes including plasmid DNA, siRNA, and therapeutic proteins with high efficiency and low cytotoxicity^6,7^. In this context, endocytotic processes and direct translocation, e.g. via pores, have been proposed to be involved in the cellular internalization^7^, but the exact mechanism by which the transport of molecules is triggered is still not fully resolved.

In recent years, the use of CPPs has gained much attention in the cosmetic industry, for topical vaccination or in clinical application. So, tremendous efforts have been made for the application of CPPs as novel transdermal delivery system providing a safer alternative over conventional oral delivery and injection^8,9^. Examples include the delivery of cyclosporine A attached to polyarginine-7 (R_7_-CsA) across the skin for the treatment of inflammatory skin diseases^10^, PEP-1-botulinum neurotoxin A fusion conjugates as cosmetic for treatment of facial wrinkles^11^, and more recently, the design of fusion proteins composed of four structural classes of CPPs with the human epidermal growth factor (hEGF) as a wound healing agent^12^.

So far, very few studies have been conducted for the use of CPPs in the application field of human skin wound healing^12–14^. Thus, to our knowledge, we report for the first time on the fusion of a well-characterized cell penetrating peptide, namely sC18, to a wound healing promoting peptide sequence Tylotoin. This latter short peptide was recently identified and isolated from the skin of the salamander *Tylototriton verrucosus*^15^. The precursor of Tylotoin was found to belong to the family of cathelicidins, which is a conserved protein family among vertebrates^15^. More precisely, Tylotoin is derived from the *C*-terminal part, such as other antimicrobial peptides, e.g. LL-37^16^ or sC18^17^, but in contrast to these, Tylotoin exhibits no antimicrobial activity^15^. However, Tylotoin was found by Mu and coworkers to possess the capability to promote wound healing similar to hEGF in a murine model^15^. Herein, these novel conjugates were evaluated upon their cytotoxicity towards keratinocytes and a cancer cell line, their cellular internalization behavior and moreover, the impact of peptides on *in vitro* wound healing was investigated.

## Results

### Peptide design and structure analysis

Herein, we synthesized chimeric peptides consisting of the wound healing promoting peptide Tylotoin^15^ and a CPP. As CPP we used sC18, which was recently developed in our group^17^, as well as a truncated version of sC18, lacking four amino acids from the *C*-terminal part, namely sC18* ^18^. The CPP was then either fused *C*- or *N*-terminally to Tylotoin resulting in 4 peptide chimeras (Table 1).

**Table 1:** Names, sequences and analytical data of peptides. All peptides are amidated at the C-terminus. For fluorescence studies they were N-terminally labeled with 5(6)-carboxyfluorescein.

Peptide	Sequence	MW_calc._ [Da]	MW_exp._ [Da]	Yield [%]	Net charge
sC18	GLRKRLRKFRNKIKEK-NH_2_	2069.55	2070.47	36	+9
sC18*	GLRKRLRKFRNK-NH_2_	1570.96	1571.44	29	+8
Tylotoin	KCVRQNNKRVCK-NH_2_	1474.81	1474.55	66	+6
Tylotoin-sC18	KCVRQNNKRVCKGLRKRLRKFRNKIKEK-NH_2_	3527.33	3527.57	74	+14
Tylotoin-sC18*	KCVRQNNKRVCKGLRKRLRKFRNK-NH_2_	3028.71	3028.80	23	+13
sC18-Tylotoin	GLRKRLRKFRNKIKEKKCVRQNNKRVCK-NH_2_	3527.33	3528.40	46	+14
sC18*-Tylotoin	GLRKRLRKFRNKKCVRQNNKRVCK-NH_2_	3028.71	3029.39	56	+13

We additionally prepared Tylotoin and the CPPs alone as control peptides.

All peptides were readily synthesized via Fmoc/*t*Bu solid-phase peptide synthesis, purified and analyzed by LC-MS methods as previously described^18,19^.

**Figure 1:**
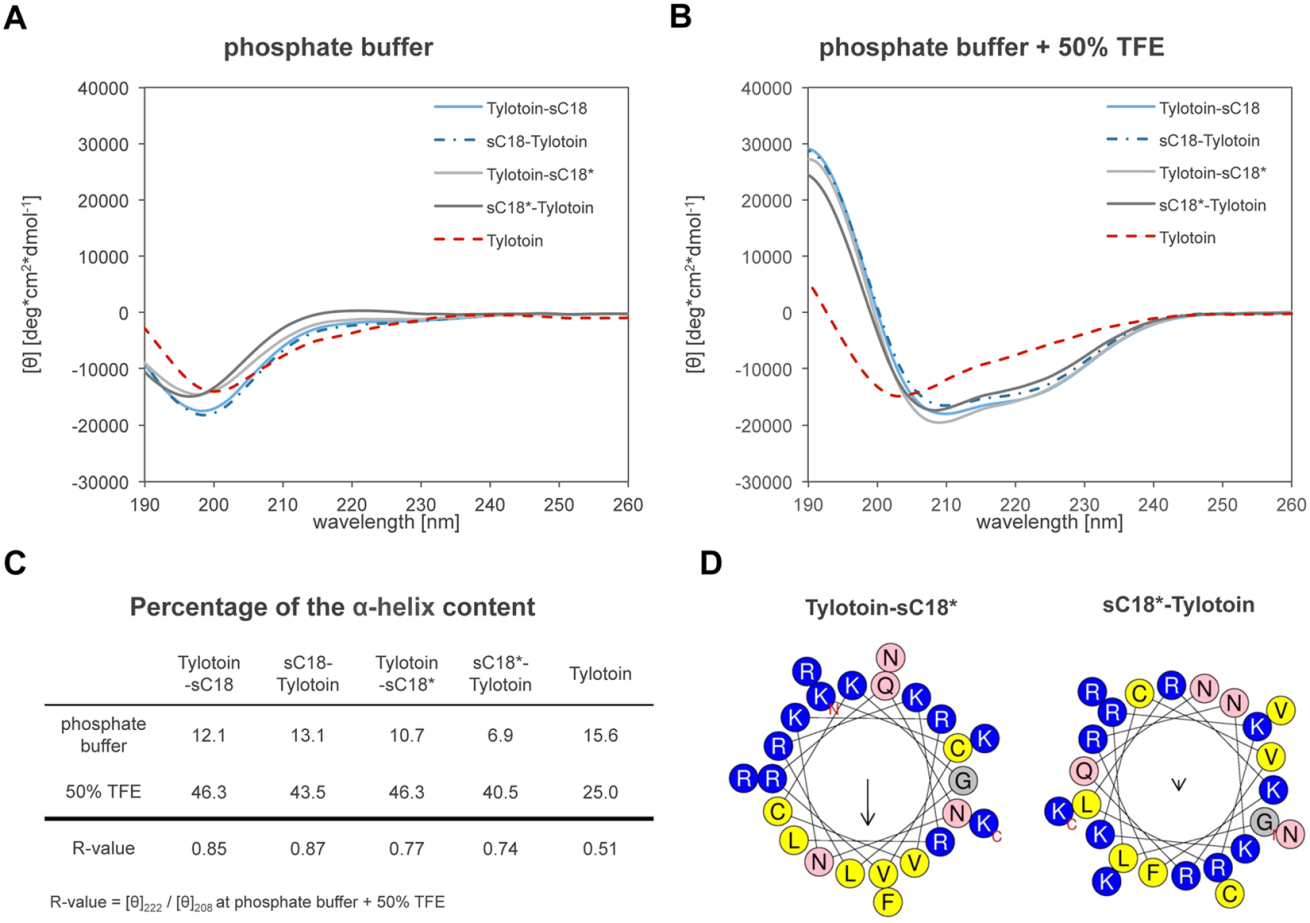
Structural characteristics of investigated peptides. CD spectra were acquired at a peptide concentration of 20 µM in 10 mM phosphate buffer, pH 7.0 (**A**) and in the additional presence of 50% TFE (**B**). The percentage of the α-helix content was calculated from the ellipticity value at 222 nm using the following formula: (% α-helix = ((([Θ_222_]−3000)⁄(−36000−3000)) × 100)^47^ (**C**). R-values represent the ratio between the molar ellipticity values at 222 and 208 nm^20^ and R = 1 defined as hallmark for a perfectly built α-helix^48^. Helical wheel projections of Tylotoin-sC18* and sC18*-Tylotoin predicted by HeliQuest (**D**).

Next, we determined the secondary structure of the peptides in the present of TFE using circular dichroism spectroscopy (Fig. 1A,B).

While all peptide conjugates as well as Tylotoin alone displayed a spectrum typical of an unordered conformation in phosphate buffer with minima around 197–200 nm, all chimeric peptides became structured in α-helix conformations with the addition of TFE. However, Tylotoin was still in an unstructured form (Fig. 1B). Since both sC18 and sC18* adopt an α-helical structure when in presence of TFE, we assumed that the attachment of the CPP to Tylotoin supports the transition to helical structures^17,18^. This was indeed supported by the estimated R-values^20^, which were significantly increased (Fig. 1C) for the chimeric peptide conjugates in the presence of 50% TFE when compared with Tylotoin alone. Moreover, the percentage of the α-helical content was slightly greater when the CPP was fused to the *C*-terminus of Tylotoin, what might be explained by taking a closer look at the helical wheel plots^21^. As representative, the helical wheel projection of Tylotoin-sC18* highlights two separate faces, one hydrophilic and one hydrophobic (Fig. 1D). In contrast, the one of sC18*-Tylotoin shows no obvious differentiation into distinct domains. Thus, we hypothesized that fusion of the CPP to the *C*-terminal part of Tylotoin leads to a better hydrophobic/hydrophilic balance allowing optimal interaction of the peptide conjugates with the amphiphilic nature of biological membranes.

### Cytotoxicity against keratinocytes

**Figure 2:**
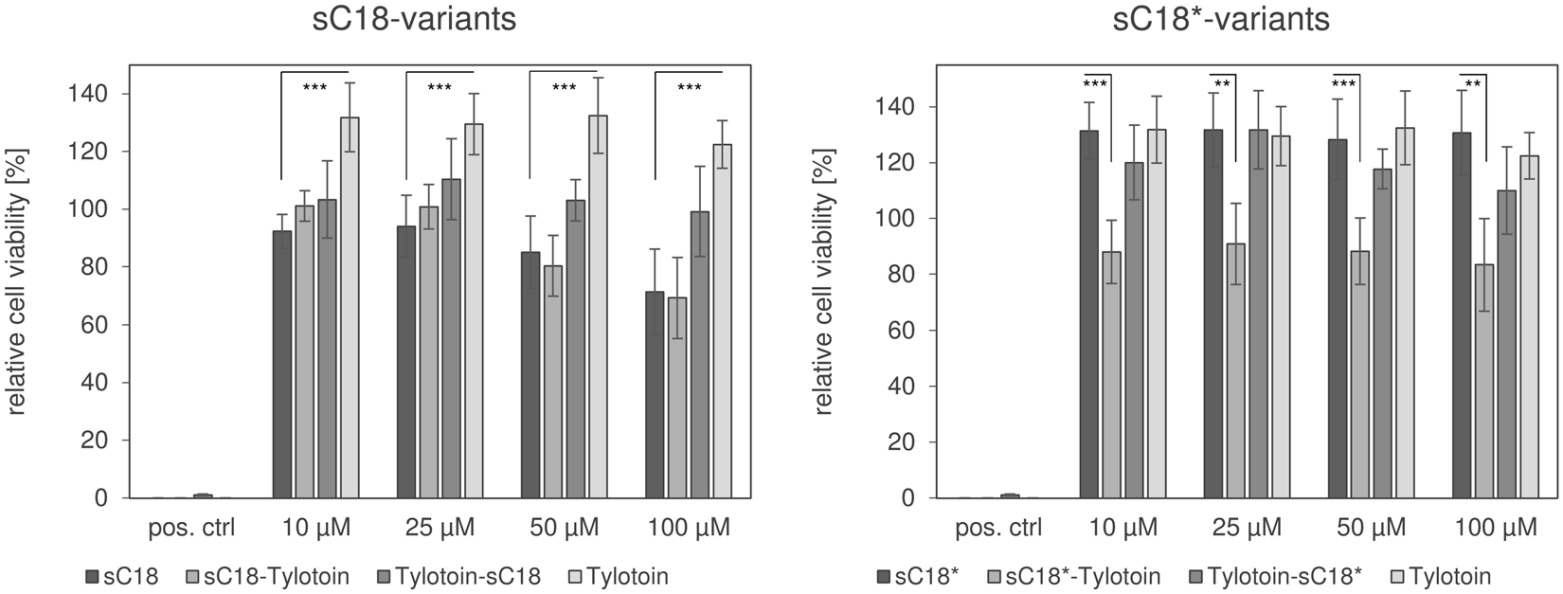
Cytotoxic profile of peptides against human keratinocytes HaCaT. Cells were incubated with indicated concentrations of peptides at 37 °C for 24 h. Cell viability of HaCaT cells was determined by MTT assay relative to untreated cells (set to 100%). As positive control, cells were treated with 70% EtOH for 10 min. Experiments were conducted in triplicate with n = 3. Error bars represent standard deviation. (**p < 0.01; ***p < 0.001).

To evaluate the novel peptides as potential wound healing agents, it is mandatory that the peptides exhibit no cytotoxic effects against human skin keratinocytes. Thus, we investigated the influence of all peptide conjugates on the cell viability towards human immortalized keratinocytes (HaCaT) (Fig. 2).

The results revealed no cytotoxic effect at all for Tylotoin, Tylotoin-sC18, sC18* alone and Tylotoin-sC18* on keratinocytes viability (Fig. 2). Moreover, Tylotoin seemed to enhance the proliferation of HaCaT cells, which agrees with previously reported data^15^. Contrarily, a slight decrease in cell viability was detected for sC18*-Tylotoin (Fig. 2B). In fact, there is a difference of about 40% between sC18* and sC18*-Tylotoin in relative cell viability. Interestingly, sC18* seems to promote cell proliferation in the same manner as Tylotoin itself and comparable to Tylotoin-sC18*, while sC18*-Tylotoin seems to negatively affect the cells, as was also seen when treating the cells with higher concentrations (>50 µM) of sC18 and sC18-Tylotoin. From these data we conclude that attachment of Tylotoin to the *N*-terminal of sC18/sC18* is more favorable. Thus, we used peptide concentrations of 10 µM for further experiments.

### Peptide internalization

**Figure 3:**
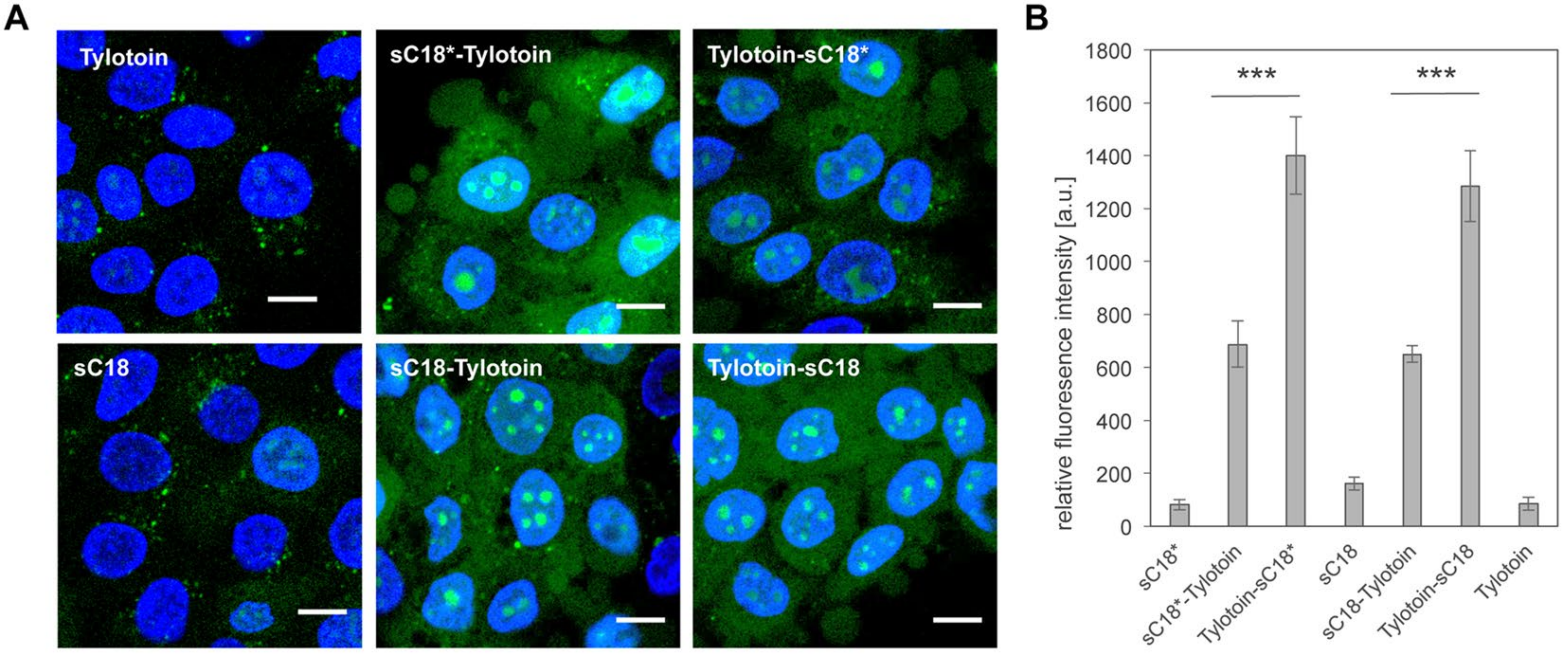
Internalization into keratinocytes. (**A**) Fluorescence microscopic images after 30 min incubation with 10 µM CF-labeled peptides. green, carboxyfluorescein-labeled peptide; blue, Hoechst 33342 nuclear stain; scale bar, 10 µm. (**B**) Corresponding flow cytometric uptake studies of 10 µM CF-labeled peptides in HaCaT cells after 30 min incubation. Experiments were conducted in triplicate with n = 3. Error bars represent the standard deviation. (***p < 0.001).

To gain some insights into the capability of peptides’ cell entry, the cellular internalization efficiency of the new peptide conjugates compared to control peptides was elucidated. Therefore, the peptides were incubated with HaCaT cells for 30 min at 37 °C (Fig. 3).

All peptides were able to internalize into keratinocytes. The control peptides were taken up to a lower extent and accumulated more into cytoplasmic vesicles, although for sC18 alone some nuclear uptake was visible. Notably, the chimeric peptides demonstrated a strong cytosolic distribution, and accumulated within the nucleoli (Fig. 3A). In addition, the intrinsic fluorescence of novel peptide chimeras measured by flow cytometry confirmed a significant greater uptake compared to control peptides (Fig. 3B). Furthermore, these observations were even more prominent when the CPP was fused to the *C*-terminus of Tylotoin.

Considering the additional proliferative effect and the favored structural arrangement of Tylotoin-sC18*, we decided to continue the following experiments with this chimera. First, we investigated further if endocytic routes can be disregarded. Therefore, HaCaT cells were incubated with Tylotoin-sC18* at 4 °C to inhibit the energy-dependent pathway^22^, and in another experiment in the presence of dynasore, where the endocytosis is totally suppressed^23,24^.

**Figure 4:**
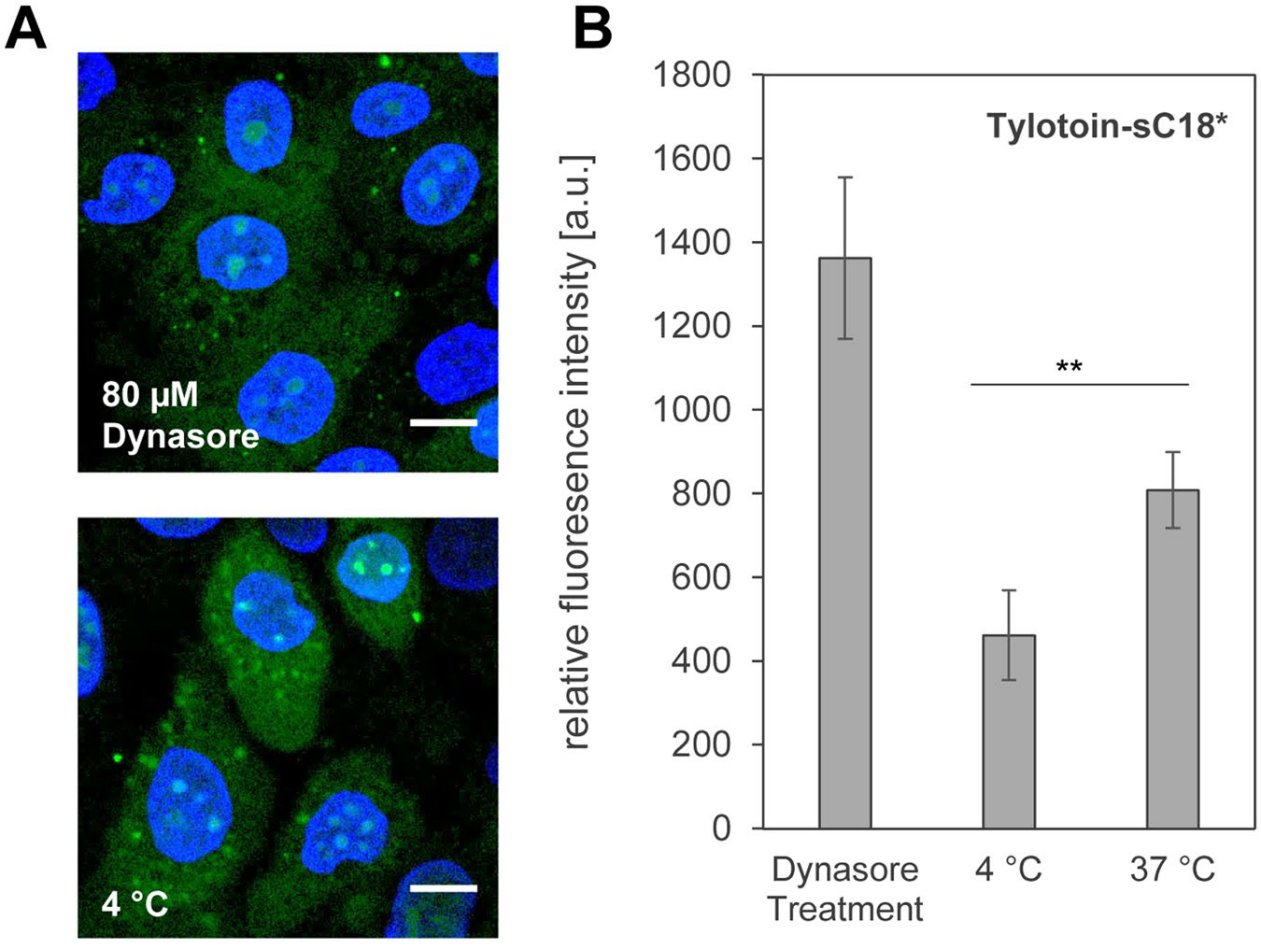
Effect of endocytosis inhibition on cellular uptake of Tylotoin-sC18* in HaCaT cells. (**A**) Cells were pretreated with 80 µM dynasore and subsequently with 10 µM CF-labeled Tylotoin-sC18* for further 30 min at 37 °C or cells were incubated with 10 µM CF-Tylotoin-sC18* for 30 min at 4 °C. green, CF-labeled peptide; blue, Hoechst 33342 nuclear stain; scale bar, 10 µm. (**B**) Corresponding flow cytometric uptake analysis of 10 µM CF-labeled peptide in HaCaT cells. (**p < 0.01) Experiments were performed in triplicate with n = 2.

The cellular uptake of CF-Tylotoin-sC18* at 4 °C was significantly decreased relative to peptides incubated at 37 °C (Fig. 4B). However, the peptide still entered the cells and was evenly distributed throughout the cytosol, with some punctuate accumulations observed (Fig. 4A). Notably, Tylotoin-sC18* was still localized within the nucleoli, approving the assumption that the peptide, to a certain extent, is also able to enter the cells by direct penetration. Interestingly, the internalization of the chimeric peptide was not suppressed in dynasore-treated skin cells, but rather showed a much higher uptake compared to the treatment at 37 °C. Considering that dynasore prevents besides dynamin, which is essential for membrane fission during clathrin- and caveolin-mediated endocytosis, also vesicle formation and fusion steps of macropinocytosis^23,24^, it can be presumed that Tylotoin-sC18* is indeed capable to internalize by direct translocation.

### Peptide-lipid interaction

**Figure 5:**
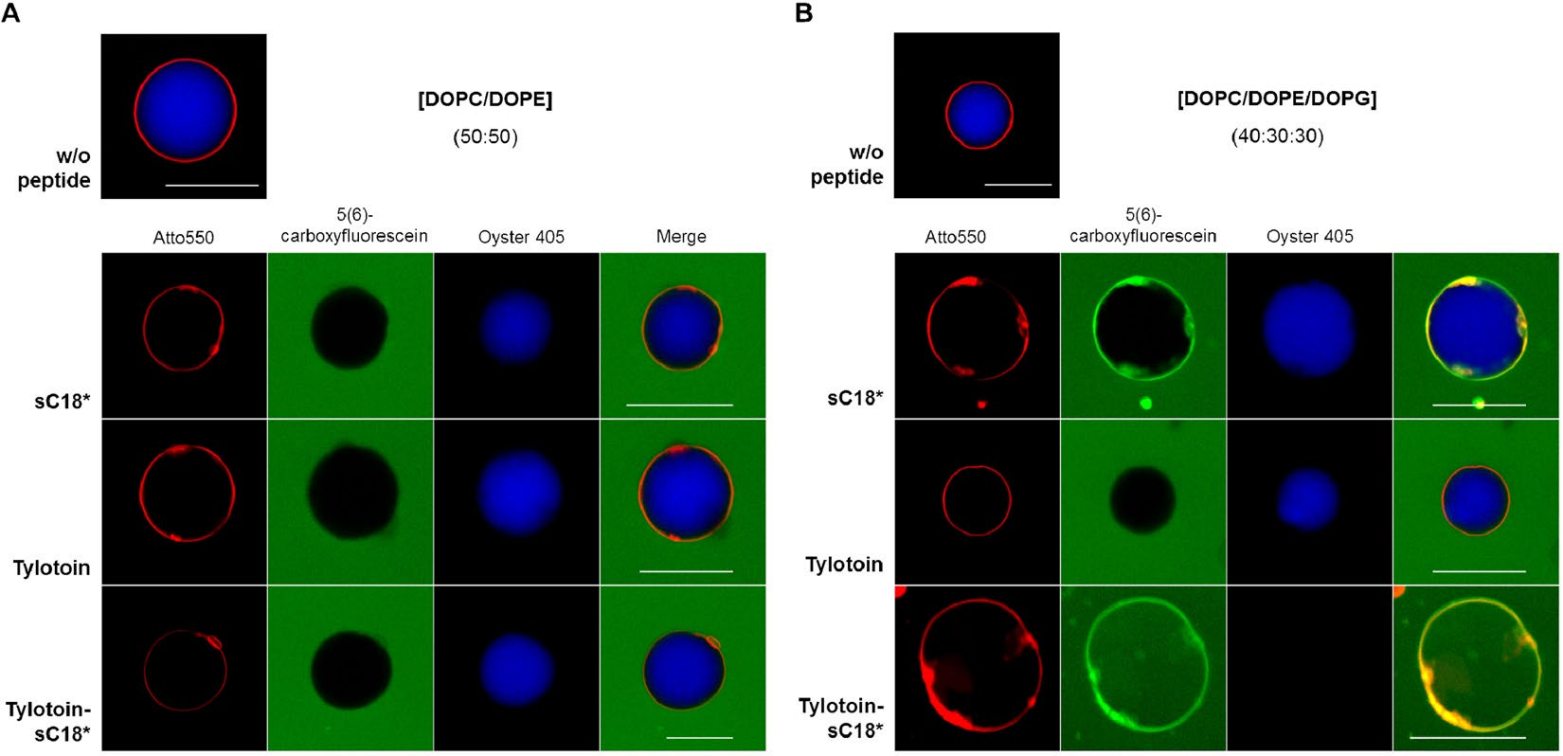
Interaction of Tylotoin-sC18* and its precursor peptide with model membranes. CF-labeled peptides (20 µM) were incubated with neutral (**A**) and negatively charged (**B**) giant unilamellar vesicles (GUVs) loaded with Oyster 405 (blue dye) for 90 min and analyzed by fluorescence microscopy. The vesicle membranes were labeled with 0.2 mol% Atto550-DOPE (red). Scale bar, 30 µm.

In a next level, we used artificial membrane systems, namely giant unilamellar vesicles (GUVs), to get an idea about the initial step of cell entry on a more molecular level. Since Tylotoin-sC18* is rich in positively charged residues (Table 1), and due to the fact that electrostatic interactions between positively charged CPPs and the negative charges within the cell membrane are of particular importance during the first stage of peptides cell entry^25–27^, GUVs composed of zwitterionic lipids (DOPC and DOPE) and with the additional incorporation of DOPG as negatively charged phospholipid were prepared to ensure better comparability. Thus, 20 µM of CF-labeled Tylotoin-sC18*, as well as CF-Tylotoin and CF-sC18* serving as controls, were incubated for 90 min with neutral GUVs (DOPC/DOPE) (Fig. 5A) and anionic GUVs (DOPC/DOPE/DOPG) (Fig. 5B).

No significant accumulation of peptides on membranes of neutral GUVs was observed, since no overlay with the red-labeled vesicle membrane could be detected (Fig. 5A). Furthermore, the influence of peptides on membrane integrity was examined by studying induced leakage of a GUV-encapsulated blue fluorescent dye. But again, no noticeable differences in the Oyster 405 signal (blue) before and after peptide addition could be revealed and thus, no obvious dye release. In contrast, when anionic GUVs were incubated with CF-labeled peptide solutions, a strong peptide signal on the membranes was detected, except for CF-Tylotoin (Fig. 5B). Furthermore, the interaction of Tylotoin-sC18* with the anionic lipid membrane resulted in a significant dye release, indicating that the membrane becomes prone to leak. Notably, Tylotoin-sC18* was thus able to completely stain the entire vesicle.

### Efficacy of Tylotoin-sC18* on *in vitro* wound healing

**Figure 6:**
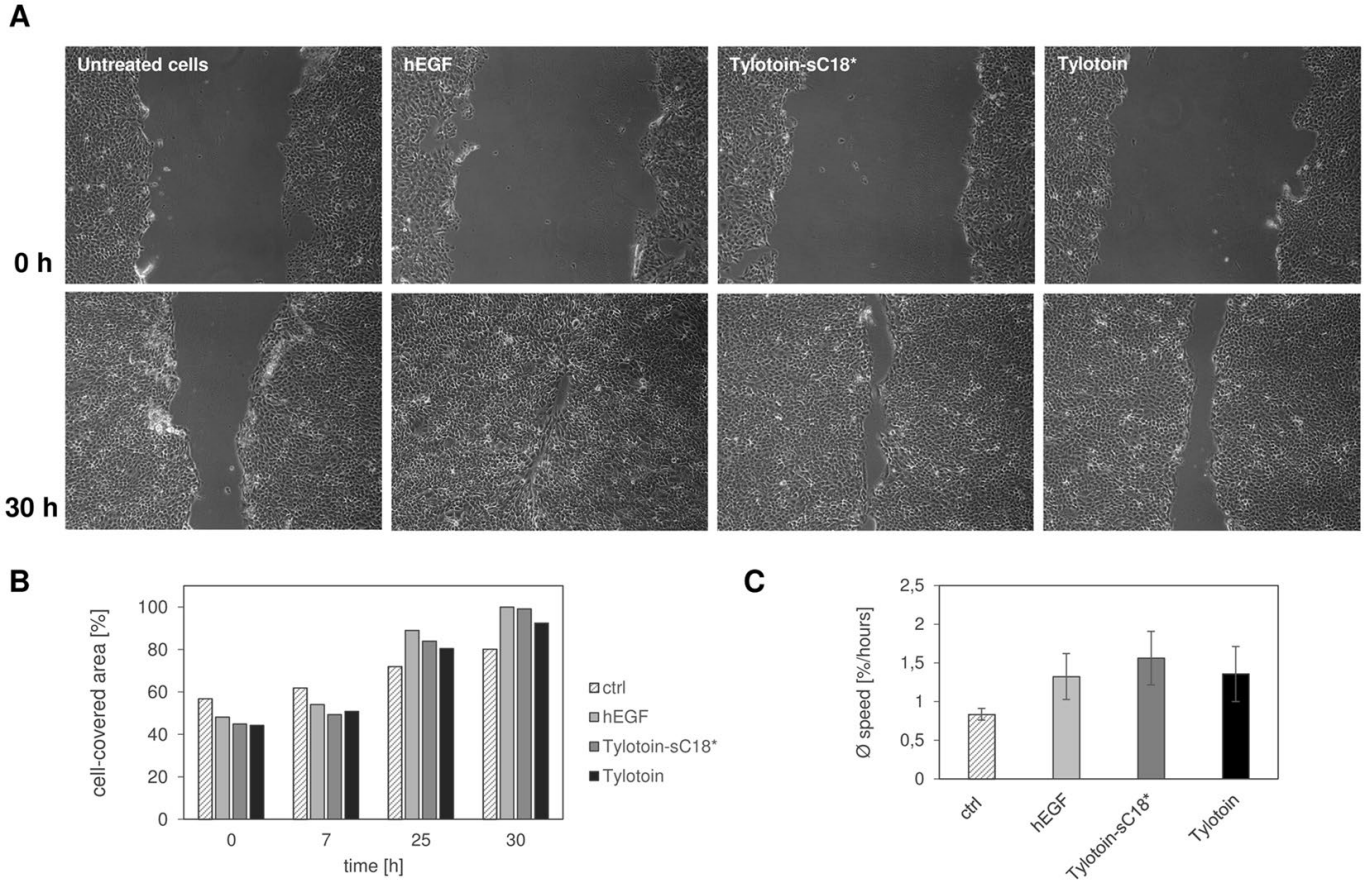
The effect of Tylotoin-sC18* on the migration of keratinocytes. Cell monolayer was wounded by scratching with a pipette tip and then treated with 13.5 µM Tylotoin-sC18*, 13.5 µM Tylotoin and 100 ng/ml hEGF, respectively. Cells without any treatment were used as the control. Wound closure was monitored at 0, 7, 25 and 30 h after peptide addition by inverted light microscopy (**A**). The percentage of corresponding cell-covered area at each time point (**B**) and the average speed (% wound closure/h) (**C**) were analyzed by Wimasis Image Analysis. The results shown are representative of at least three independent experiments.

Keratinocytes are the predominant cell type of the epidermis protecting it from external agents and pathogens^28^. Upon injury of the skin, migration and proliferation of keratinocytes represent a crucial step in the re-epithelization phase of the wound healing process^28–32^. Mu *et al*. already showed that Tylotoin activates the migration of keratinocytes, which resulted in the promotion of skin wound healing^15^, and thus, we were interested whether the attachment to a CPP has adverse effects on its wound healing promoting activity. We used a scratch wound closure assay to investigate the healing activity of Tylotoin-sC18* on HaCaT cells (Fig. 6).

The migration of keratinocytes into the wounded area was distinctly increased in the presence of 13.5 µM Tylotoin-sC18* 30 h after scratching and similar with the human epidermal growth factor (hEGF) (Fig. 6A,B). From this we propose that the novel designed peptide conjugate had comparable migration efficiency with Tylotoin and hEGF, suggesting that the CPP fused to Tylotoin did not cause any loss in its activity. Of note is that the ability of Tylotoin-sC18* to enhance migration of keratinocytes seemed to be similar and presumably slightly increased compared to Tylotoin alone, since the average speed of the cell-covered area per hour was slightly accelerated for Tylotoin-sC18* (Fig. 6C) (although this effect was not statistically significant). Moreover, since wound closure results from both cell migration and proliferation, the wound healing assay was also performed in the presence of mitomycin C, which is known to inhibit mitosis of the cells and thus allows distinguishing migration from proliferation.

**Figure 7:**
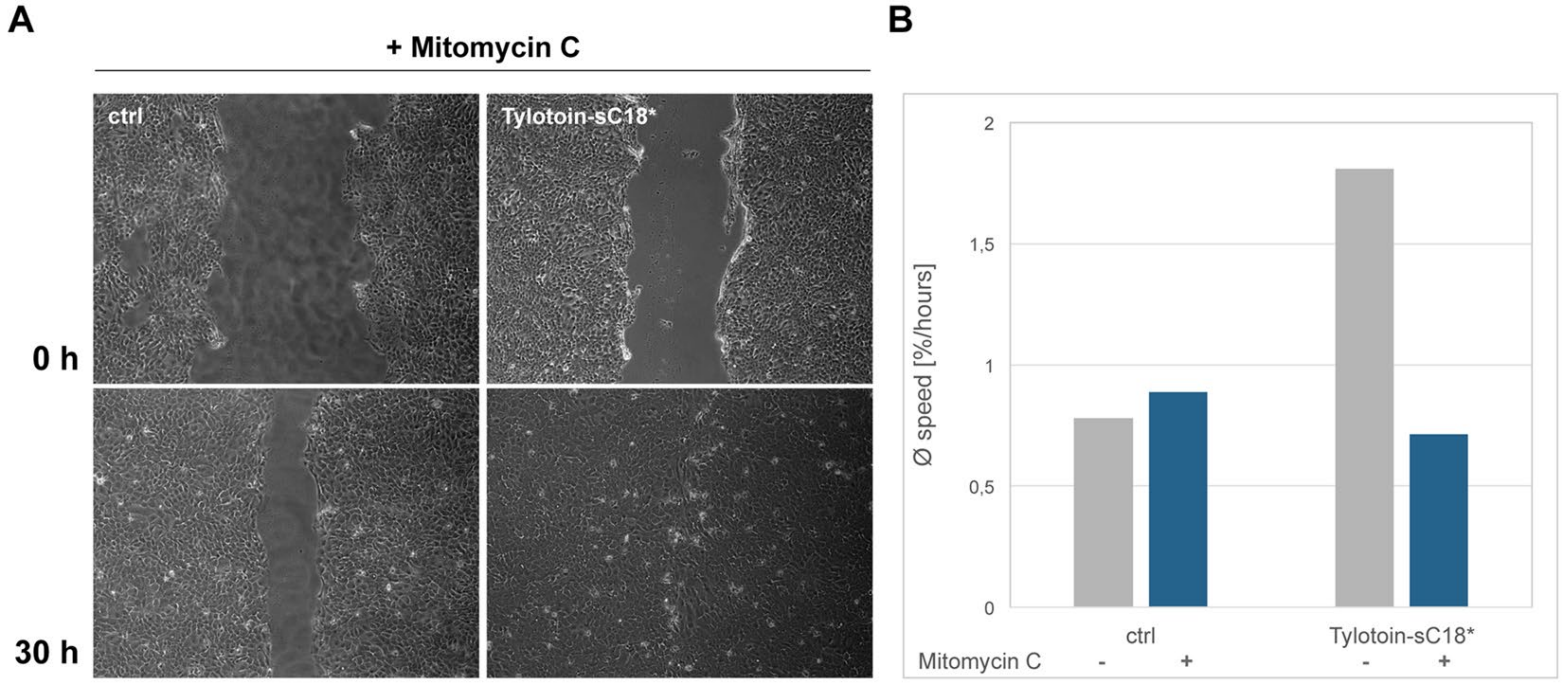
Influence of mitomycin C on Tylotoin-sC18*-induced wound closure. Confluent monolayer of immortalized HaCaT cells were treated with 20 µM mitomycin C for 90 min prior to scrape formation to avoid cell proliferation. Wound closure in the presence of 13.5 µM peptide, or even maintenance medium was monitored after 0, 7, 25 and 30 h, respectively, by inverted light microscope. Shown are time-points 0 and 30 h (**A**). Corresponding mean speed (% wound closure/hours) of wound healing was calculated by Wimasis Image Analysis (**B**).

Data shown in Fig. 7A let conclude that the wound healing rate of Tylotoin-sC18* is higher than in the control group, since a larger number of cells migrated into the wounded gap in the presence of chimeric conjugate, which resulted in complete wound closure. But taking a closer look on mean speed, expressed as cell-covered area per hour, it was found that the presence of mitomycin C remarkably suppressed the cell migration ability of Tylotoin-sC18* (Fig. 7B). These results might illustrate that besides migration also proliferation of HaCaT cells contributed to the wound-healing effect induced by Tylotoin-sC18* as well.

### Antimicrobial activity

Recently, it was shown by our group that the cell penetrating peptide sC18 revealed moderate antimicrobial activities, which could be further increased by conjugation with imidazolium salts^33^. Moreover, it has been demonstrated that other natural peptides, which act as important modulators in wound healing, are often characterized by additional beneficial features e.g. antimicrobial activities against a broad spectrum of bacteria^34,35^. However, Tylotoin alone did not show any antimicrobial activity^15^. Therefore, it was particularly interesting whether the combination of both, Tylotoin and sC18*, would lead to an efficient peptide conjugate with antimicrobial activity. Thus, we determined the potency of Tylotoin-sC18* against representatives of a gram-positive and -negative bacterial strains.

**Figure 8:**
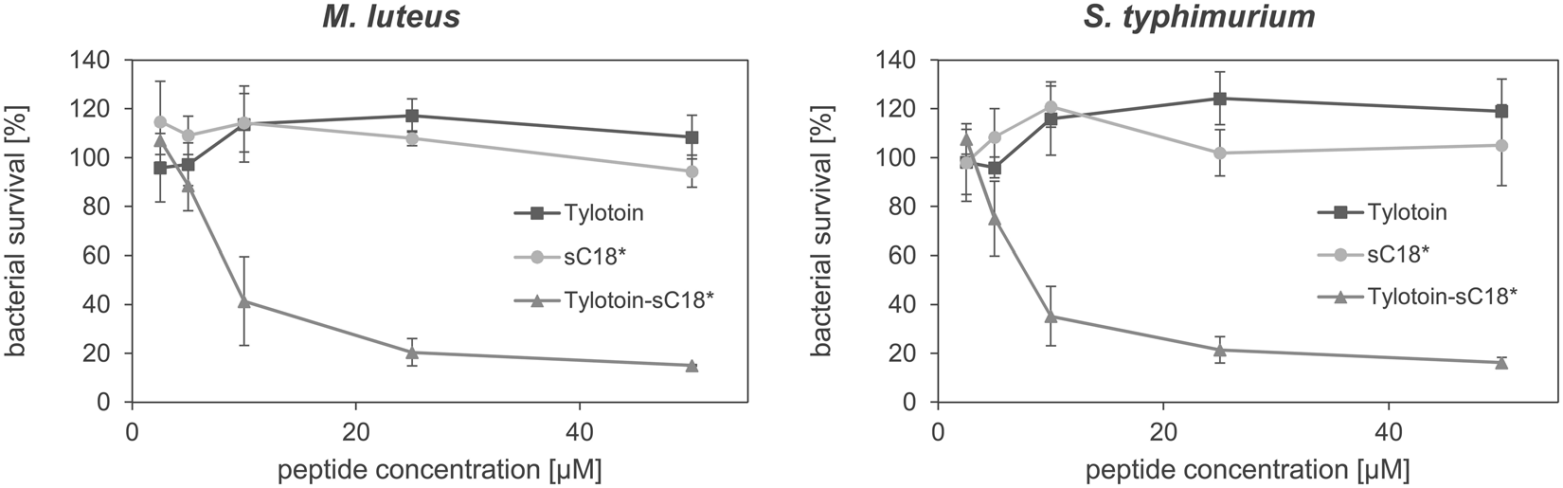
Antimicrobial effect of peptides against gram-positive and gram-negative bacteria. M. luteus and S. typhimurium were incubated with indicated concentrations of peptides at 37 °C. After 6 h of incubation, bacterial survival is expressed as percentage with respect to water-treated bacteria using INT assay. Treatment with 10 µM chloramphenicol served as positive control. Experiments were achieved in triplicate with n = 2. Error bars represent standard deviation.

Unlike other cathelicidins^36^, sC18* and Tylotoin alone showed no antimicrobial activity against the gram-positive bacteria *Micrococcus luteus* and the gram-negative bacterial strain *Salmonella typhimurium* (Fig. 8). Fortunately, Tylotoin-sC18* revealed a strong activity against the gram-positive bacteria (MIC value of 13.47 µM), thus relative bacterial survival was diminished by about 60% at 10 µM and decreased further to 15% of living bacteria at a peptide concentration of 50 µM. Similar results were obtained when the gram-negative bacteria was incubated with peptides for 6 h (Fig. 8, right panel). Here again, Tylotoin-sC18* turned out to exhibit very promising effects against *S. typhimurium* with an MIC value of ~12.64 µM, while Tylotoin and sC18* alone showed no antimicrobial activities. Furthermore, the positive control chloramphenicol, a commonly used antibiotic^37^, reduced the cell viability by around 50% at a concentration of 10 µM (data not shown) which was overall less active compared to Tylotoin-sC18*, highlighting again its high antimicrobial potential.

### Preferential interaction with cancer cells

Since a large body of literature reported on peptides with dual antimicrobial and anticancer properties, we investigated the behavior of our novel peptide conjugate towards a cancer cell line, in particular HeLa cells^38^.

**Figure 9:**
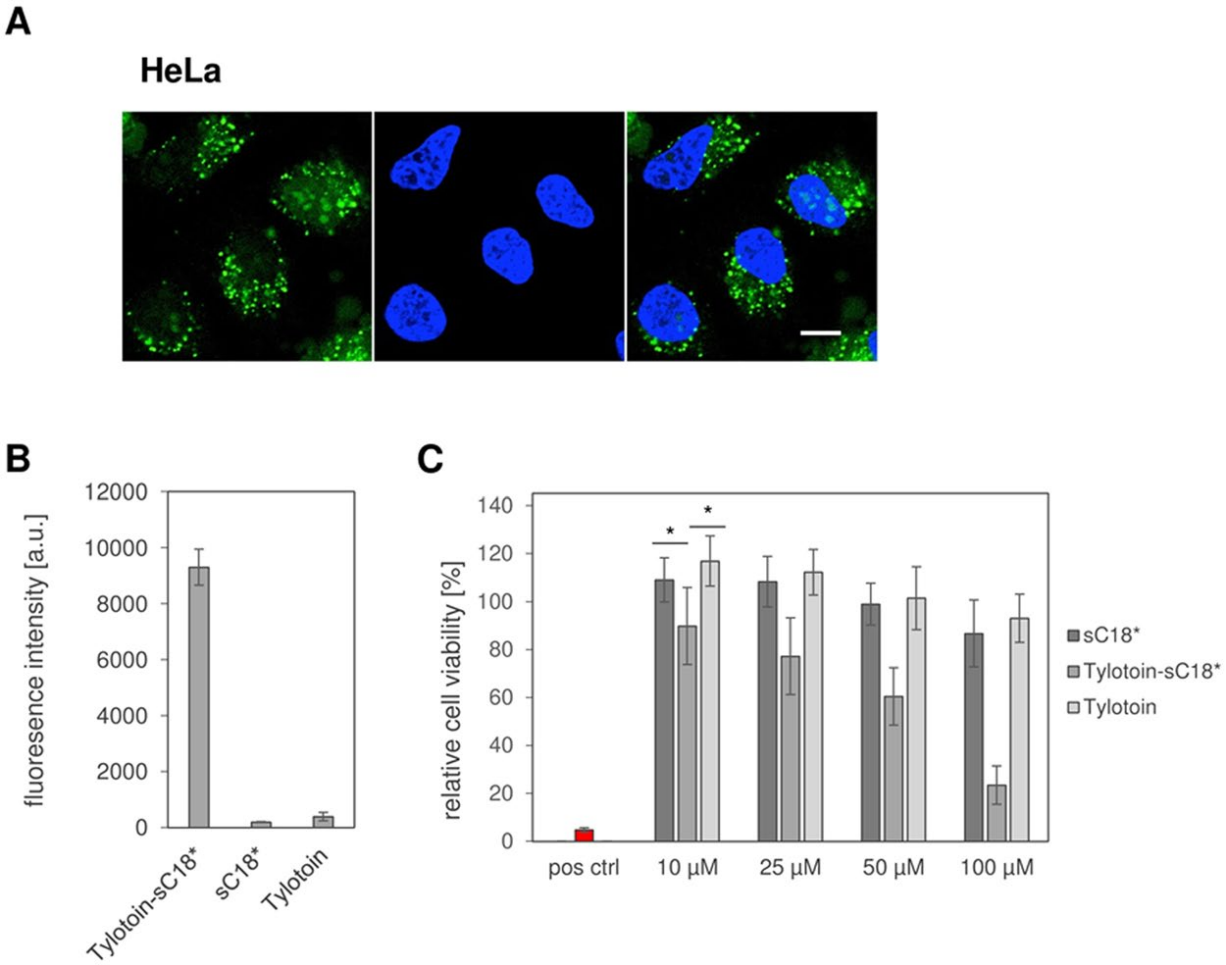
The efficacy of Tylotoin-sC18* towards cancer cells. Microscopic image of HeLa cells treated for 30 min with CF-labeled Tylotoin-sC18* (10 µM, green) at 37 °C (**A**). Quantification of the cellular uptake of 10 µM CF-labeled peptides for 30 min at 37 °C by flow cytometry (**B**). Cell viability of HeLa cells after treatment for 24 h with Tylotoin-sC18*, sC18* and Tylotoin, respectively (**C**). Cells treated with 70% ethanol served as positive control. Data are normalized to untreated cells (100% cell viability). Experiments were performed in triplicate with n = 3. Error bars represent standard deviation. (*p < 0.05).

CF-labeled Tylotoin-sC18* was characterized by an extremely high uptake in HeLa cells compared to control peptides, which was accompanied by vesicular distribution pattern as well as accumulation into the nucleus (Fig. 9A,B). Furthermore, cell viability assays demonstrated that after 24 h incubation, HeLa cells remained unharmed when treated with sC18* and Tylotoin alone (Fig. 9C). Interestingly, Tylotoin-sC18* induced a steady decrease of cell viability, which was already significant at lower concentrations and might probably hint to additional anticancer activity of this peptide.

## Discussion

In this study, we evaluated the biological activities of novel peptide conjugates combining for the first time, a cell penetrating peptide and a wound healing promoting sequence to one molecule. The structural analysis revealed that the presence of the CPP supported the formation of secondary structures like α-helices for the chimeric peptides in a membrane-mimetic environment. Quite recently, we additionally demonstrated that fusion of sC18* to nuclear-targeting sequences contributed to a similar conformational change approving the assumption that the presence of sC18/sC18* is responsible for this observation^39^. Moreover, the formation of such favored amphiphilic α-helices is one key factor for efficient and optimal peptide/lipid interaction crucial for the initial step of cell entry^40^. Consequently, the cellular internalization into skin cells was drastically increased compared to control peptides. However, the uptake of Tylotoin into keratinocytes, moreover into the nucleoli, was herein reported for the first time. This observation might be due to the fact that we synthesized Tylotoin as a *C*-terminal acid amide and without the disulfide bridge. Thus, Tylotoin probably benefits from this lacking negative charge leading to the observed cellular uptake. Nonetheless, for other amphibian skin peptides, e.g. Esc(1–21)^41^ or temporin^42^, internalization into the keratinocytes cytoplasm was already published. To go into deeper detail, we further studied the influence of endocytosis inhibition, since we observed cellular punctate distributions characteristic for endocytosis^43^. At lower temperature, the cellular uptake of Tylotoin-sC18* was significantly diminished compared to incubation at 37 °C. Surprisingly, in the presence of dynasore, an endocytosis inhibitor, the internalization efficiency was strongly improved leading to the final assumption that the novel conjugate penetrated the cells by direct translocation as well. These findings are in agreement with the fact that shutting down one uptake route might induce the cellular translocation through another pathway that is normally suppressed^7^. In addition, we observed that vesicular structures were much more pronounced after a longer incubation time (Fig. S1). Moreover, the peptide signal did not overlay with any cellular organelle tracker, e.g. endosomes, lysosomes or mitochondria (data not shown), letting us conclude that Tylotoin-sC18* might be intracellularly aggregated over time. Overall, since model membrane systems are not affected by endocytotic processes, we hypothesize that Tylotoin-sC18* is indeed able to directly penetrate the cell membrane through membrane destabilization effects.

Furthermore, we revealed a higher internalization into the human cancer cell line HeLa, which is in accordance with strong interaction with the anionic membrane of giant unilamellar vesicles. It is known that cancer cells possess a more negatively charged outer leaflet than healthy eukaryotic cells^26^, thus, it might be possible that the novel conjugate is more selective against cancer cells. The ability of Tylotoin-sC18* to interact with negatively charged moieties might derive from its positive net charge accompanied by its amphiphilic nature of the adopted α- helix.

Further, we showed that fusion of the cell penetrating peptide sC18* to Tylotoin did not result in a loss of Tylotoins wound healing promoting ability. On the contrary, Tylotoin-sC18* did further improve this property, as illustrated by the capability of the conjugate to promote *in vitro* wound closure by inducing migration and proliferation of keratinocytes. Additionally, Tylotoin-sC18* exhibited improved antimicrobial activity against representatives of gram-positive and -negative bacterial strains, which is quite impressive considering that efficient wound healing is often hampered by bacterial infections^44^.

## Conclusion

Taken together, our results on the antimicrobial and wound healing activities suggest that Tylotoin-sC18*, a chimera consisting of a wound healing promoting peptide and a cell penetrating peptide, might provide a novel peptide candidate for the development of wound-healing promoting agents in infected wounds. Moreover, the covalent attachment of Tylotoin to the *N*-terminus of the cell penetrating peptide sC18* resulted in a favored amphiphilic character probably crucial for the detected higher cellular uptake. The strong interaction with negatively charged lipid moieties of model membranes as well as cancer cells corroborated the importance of anionic membrane components for lipid-membrane interaction. Further, Tylotoin-sC18* appears to penetrate the cancer cell line more efficiently than the skin cells leading to the assumption that this chimera exhibited additionally a certain selectively towards cancer cells. This is not uncommon since a number of antimicrobial peptides encountered in nature show anticancer efficacy without any significant cytotoxicity activity as well^38^. Finally, these features highlight that Tylotoin-sC18* might be established as a topical agent for infected wound treatment.

## Materials and Methods

### Cell lines and bacterial strains

HaCaT (immortalized human keratinocytes) and HeLa (human cervix carcinoma) cells were cultured in sterile 10 cm petri dishes at 37 °C in a humidified atmosphere of 5% CO_2_. HaCaT cells were maintained in DMEM containing 4,500 mg/l glucose (DMEMg), 2 mM L-Gln and 10% FBS (fetal bovine serum). HeLa cells were grown in RPMI 1640 supplemented with 4 mM L-Gln and 10% FBS. Bacteria used were *Micrococcus luteus* (DSM 20030) and *Salmonella typhimurium* (TA 100). Mueller Hinton (MH) broth agar plates were used for maintenance und MH broth medium was used to carry out antimicrobial activity studies.

### Peptides

Peptides were synthesized on Rink amide resin by automated solid-phase peptide synthesis (SPPS) on a multiple Syro II peptide synthesizer (MultiSynTech, Witten, Germany) following Fmoc/*t*Bu-strategy utilizing a double-coupling procedure and *in situ* activation with Oxyma/DIC. The synthesized peptide conjugates were purified using preparative reversed phase HPLC and analyzed by analytical RP HPLC ESI-MS according to refs^39,45^.

### Circular dichroism (CD) spectroscopy

CD spectra were recorded from 180 nm to 270 nm in 0.5 nm intervals at 20 °C using a JASCO J-715 spectropolarimeter in an N_2_ atmosphere. The CD spectra were measured using a 1 mm quartz cuvette and the instrument parameters were set as follows: sensitivity, 100 mdeg; scan mode, continuous; scan speed, 50 nm/min; response time, 2 sec and bandwidth, 1.0 nm. Peptides were dissolved in 10 mM potassium phosphate buffer (pH 7.0) containing either 0 or 50% (v/v) trifluoroethanol (TFE) to a final peptide concentration of 20 µM.

### Resazurin-based cell viability assay

For a resazurin-based cell viability assay, HeLa cells were seeded onto a 96-well plate, grown to 70–80% confluency and incubated with various concentrations of peptides in serum-free RPMI growth medium for 24 h under standard growth conditions. For the positive control, cells were treated with 70% EtOH for 10 min. After washing with serum-free medium, resazurin solution (10% in serum-free media, v/v) was incubated with the cells for 1 h at 37 °C. Subsequently, cell viability was determined relative to untreated cells by measurement of the resorufin product at 595 nm (λex = 550 nm) on a Tecan infinite M200 plate reader.

### Tetrazolium-based cell viability assay

The toxic effect of the investigated peptides on HaCaT cells was evaluated using the [3(4,5-dimethylthiazol-2yl)2,5-diphenyltetrazolium bromide] (MTT) colorimetric method. MTT is a tetrazolium salt which is reduced to a purple colored formazan product by mitochondrial reductases, present only in metabolically active cells. Keratinocytes were seeded onto a 96-well microtiter plate at a density of 8 × 10^4^ cells per well in DMEMg supplemented with 2 mM L-Gln and 2% FBS. After reaching 70–80% confluency, the medium was removed and cells were incubated at 37 °C for 24 h with peptides at different concentrations ranging from 10 to 100 µM (in serum-free medium). After 24 h of incubation, 10 µl of MTT (in PBS, 0.45 mg/ml final) were added to each well. After 4 h incubation, the supernatants were removed and the formazan crystals were dissolved by adding 100 μl of dimethyl sulfoxide (DMSO). For the positive control, cells were treated with 70% EtOH for 10 min. The absorption was measured at 570 nm and at a reference wavelength of 630 nm using Tecan infinite M200 plate reader.

### Antimicrobial activity

Antimicrobial activity of peptide conjugates against gram-positive and gram-negative bacteria was conducted using the p-iodonitrotetrazolium-chloride (INT) violet assay as previously described^33^. INT is a tetrazolium-based reagent which is converted to a pink formazan dye in the presence of metabolically active bacteria.

In brief, *M. luteus* and *S. typhimurium* were cultured in MH broth overnight at 37 °C while mild shaking (180 rpm). Overnight-cultured bacteria were added into fresh medium, grown to an optical density of 0.7 at 600 nm and 10 µl bacterial solution were added in triplicate to wells of a 96-well plate (BD Falcon, USA). The peptides used at various concentrations and 180 µl of MH medium were added to each corresponding well and incubated for 6 h at 37 °C. 10 µM chloramphenicol served as positive control and H_2_0 was used as negative control. After incubation time, 10 μl of the freshly prepared INT solution (1 mg/ml in DMSO) were added to each well and incubated for further 30 min at 37 °C. The viability of bacteria relative to untreated bacteria was determined by measuring the absorption at 540 nm on a Tecan infinite M200 plate reader.

### Confocal microscopic uptake studies

Cells were seeded in a µ-slide 8-well (Ibidi) plate (HaCaT 90,000; HeLa 45,000) and grown to 70–80% confluency. Then, the cells were incubated with CF-labeled peptides in serum-free medium for the requested time at either 4 °C or 37 °C as indicated. The nuclei were stained for 10 min with 0.6 µl Hoechst 33342 nuclear dye (bisbenzimide H33342, 1 mg/ml in H2O, sterile filtered) prior to the end of peptide incubation. Finally, the solutions were removed and cells were treated for 30 s with trypan blue (150 µM in 0.1 M acetate buffer, pH 4.15) for quenching external fluorescence. After washing twice with PBS, 300 µl of FBS-containing growth medium were added to each well. Images were taken by using a Nikon Eclipse Ti confocal laser scanning microscope equipped with a 60x oil-immersion objective (N.A. 1.4, Plan APO VC, Nikon). Images were recorded with Nikon EZ-C1 software and adjusted equally with ImageJ software.

For treatment with endocytosis inhibitor, 80 µM of dynasore (in serum-free medium, Sigma-Aldrich) was added to the cells. After 1 h of incubation at 37 °C, CF-labeled peptide solution (in serum-free medium, 10 µM final) was added and incubated for further 30 min. Afterwards, the cells were handled as described above.

### Flow cytometric uptake studies

Cells were seeded in triplicate in a 24-well plate (HaCaT 370,000; HeLa 170,000) and grown to 70–80% confluency under normal growth conditions. After incubation at 37 °C for 30 min with CF-labeled peptides in serum-free medium, the cells were washed twice with PBS, trypsinized and resuspended in indicator-free medium. Analyses were performed on a Guava easyCyte flow cytometer (Merck, Darmstadt, Germany). Cellular autofluorescence was subtracted.

For inhibition of endocytosis, cells were treated with CF-labeled peptide solutions for 30 min at 4 °C or cells were pretreated with 80 µM of dynasore (in serum-free medium) for 1 h at 37 °C. Finally, it was proceeded as described above.

### Preparation of giant unilamellar vesicles (GUVs)

GUVs were generated as previously described^33,46^. In brief, super low melting agarose (1%, w/v) was coated on a clean glass slide and dried on a hot plate (~50 °C) for 30 min. Afterwards, 10 µl of the respective lipid solution was spread on the agarose film and dried *in vacu*o for at least 1 h to remove residual chloroform. To visualize the membranes, the lipid solution was prior doped with 0.2 mol% Atto550-labeled DOPE. Then, a seal ring was placed onto the lipid-coated areas on the slide to obtain two sealed chambers. For the preparation of GUVs encapsulating the blue dye Oyster 405 (Luminaris GmbH, Münster, Germany), dextran buffer (HEPES,10 mM (pH 7.4); KCl 50 mM; NaCl 50 mM; dextran 1 mg/ml (from *Leuconostoc spp*., 6 kDa) containing additionally 5 μM of Oyster 405 was added to the hybrid film. The glass slide was then left in the dark for 2 h to allow hydration and swelling of the lipids. To harvest the GUV suspension, the glass slide was gently tilted in all directions to detach the liposomes from the surface. The giant liposomes were then stored in LoBind tubes (1.5 ml, Eppendorf, Hamburg, Germany) at RT and were used within three days.

### Fluorescence microscopic analysis of GUVs

Microscopic studies with GUVs were performed as recently described by our working group^19,33^. GUVs loaded with the membrane-impermeant fluorophore Oyster 405 were prepared as described above. To remove untrapped Oyster 405, liposomes were centrifuged two times at 14,000 × g for 10 min at RT. A 40 μl aliquot of the GUV solution was diluted in 50 μl of the respective buffer without Oyster 405 and was then transferred into a tissue culture vessel (FlexiPERM slide, 8 wells, Sarstedt, Germany). CF-labeled peptides diluted in dextran buffer were added to the outer solution of GUVs at a final concentration of 20 μM. GUV−peptide interaction was analyzed using a confocal laser scanning system (Nikon D-Eclipse C1) consisting of an inverted microscope (Nikon Eclipse Ti) equipped with a 20x objective (NA 0.45, Plan Fluor; Nikon). Microscope pictures were recorded in 16-bit grayscale, pseudo-colored in red (channel 1), green (channel 2), and blue (channel 3) followed by processing with ImageJ.

### *In vitro* scratch wound closure assay

The ability of peptides to promote the migration of keratinocytes was studied using an *in vitro* wound healing migration assay. Briefly, HaCaT cells suspended in DMEMg containing 10% FBS were seeded in 12-well plates and grown until they reached confluence. Next, cells were starved in serum-free media for 16 h at 37 °C in a humidified atmosphere of 5% CO_2_. For inhibitor studies, mitomycin C (20 μM final, in serum-free medium) to prevent cell proliferation were added 90 min prior to scratch generation. After this, an artificial wound was created across cell monolayers by performing a vertical scratch with a sterile 200 µl pipette tip resulting in a cell-free area of approximately 100–200 µm. After washing twice with PBS to remove cell debris, cells were treated with 13.5 µM peptides in serum containing medium. Untreated cells served as negative control and human epidermal growth factor hEGF (in serum-free medium, 5 ng/ml final) as positive control. Scratches were photographed immediately after peptide treatment and 7, 25 and 30 h after peptide addition using an inverted microscope at 10x magnification (N.A. 0.3, Plan Fluor, Nikon). Same image fields were captured and the cell-covered areas and migration speed were estimated by Wimasis Image Analysis. Experiments were repeated >3 times and representative pictures are shown.

### Statistical Analysis

All data are presented as means ± standard deviation from at least two independent experiments. Student’s t-test was carried out for the comparison between two groups. Statistical significance was set at *P* < 0.05.

## Electronic supplementary material

Supplementary Information
