# Supplementary material for: Design of a novel cell-permeable chimeric peptide to promote wound healing
Source: Sci Rep. 2018 Nov 2;8:16279. doi: 10.1038/s41598-018-34684-1 (PMC6214915; doi:10.1038/s41598-018-34684-1)
Supplement: Supplementary file 1 — Supplementary Information [file 41598_2018_34684_MOESM1_ESM.docx]

**Supplementary Information**

**Design of a novel cell-permeable chimeric peptide to promote wound healing**

Mareike Horn ^1^ and Ines Neundorf ^1,*^

Address: ^1^Department of Chemistry, Biochemistry, University of Cologne, Zuelpicher Str. 47a, 50674 Cologne, Germany


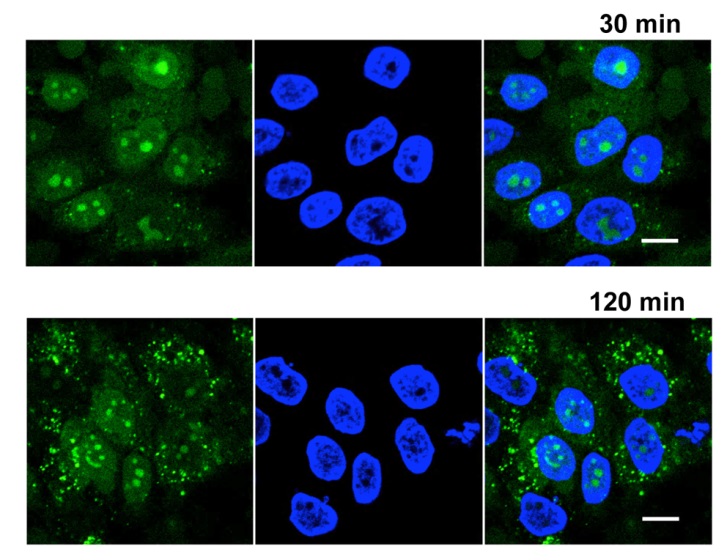


**Figure S1. Internalization of Tylotoin-sC18* into keratinocytes over time.** Fluorescence microscopic images after incubation with 10 µM CF-labeled Tylotoin-sC18 for 30 min and 120 min, respectively**.** Green, carboxyfluorescein-labeled peptide; blue, Hoechst 33342 nuclear stain; scale bar, 10 µm. After 30 min, the peptide is evenly distributed throughout the cell, including accumulation within the nuclei/nucleoli. After 120 incubation, the peptide seems to aggregate within the cells.
